# Genome sequence of a carbapenem-resistant *Salmonella enterica* serovar Typhimurium strain 142109

**DOI:** 10.1128/mra.00453-25

**Published:** 2025-06-18

**Authors:** Linwan Zhang, Yuling Xiao, Yu Feng, Yanling He, Zhiyong Zong

**Affiliations:** 1 Center of Infectious Diseases, West China Hospital, Sichuan University 12530, Chengdu, China; 2 Department of Clinical Research Management, West China Hospital, Sichuan University 12530, Chengdu, Sichuan, China; 3 Department of Laboratory Medicine, West China Hospital, Sichuan University 12530, Chengdu, China; 4 Laboratory of Pathogen Research, West China Hospital, Sichuan University 12530, Chengdu, Sichuan, China; 5 Division of Infectious Diseases, State Key Laboratory of Biotherapy, Chengdu, China; University of Maryland School of Medicine, Baltimore, Maryland, USA

**Keywords:** *Salmonella enterica*, carbapenem resistance, feces

## Abstract

We report the draft genome sequence of a carbapenem-resistant *Salmonella enterica* serovar Typhimurium strain isolated from a fecal sample. The strain harbors the carbapenemase gene *bla*_NDM-5_. The assembled genome consists of 122 contigs, totaling 5,191,762  bp, with a GC content of 51.86%.

## ANNOUNCEMENT

*Salmonella enterica* serovar Typhimurium is a common Gram-negative pathogen and a major causative agent of typhoid fever. Infected individuals typically present with acute bloodstream infections accompanied by fever, diarrhea, and other systemic symptoms (1, 2). Although quinolones remain first-line therapy, antimicrobial resistance, particularly to carbapenems, has surged over the past decade (3, 4). As carbapenems are critical antibiotics, here we report a clinical *Salmonella enterica* serovar Typhimurium strain exhibiting carbapenem resistance, aiming to provide a critical genomic resource for identifying transmission clusters and informing targeted infection control of this clinically significant pathogen. This study was approved by the Ethical Committee of West China Hospital, with informed consent being waived.

In 2024, a fecal sample from a patient with diarrhea was streaked onto *Salmonella* Chromogenic Agar Plate (Hopebiol, Qingdao, China) and incubated aerobically at 37°C for 18 hours at West China Hospital to identify the causative pathogen. A single purple colony, designated as 142109, was inoculated into Luria–Bertani broth and incubated under the same conditions overnight. Genomic DNA was extracted using QIAamp DNA Blood Mini Kit (Qiagen, Hilden, Germany), quantified with Qubit dsDNA HS Assay (Thermo Fisher Scientific, MA, USA), and its integrity was verified by electrophoresis. A 350 bp insert library was constructed with NEBNext Ultra II DNA Library Prep Kit (New England Biolabs, Ipswich, MA, USA), and fragment size distribution was assessed on Agilent 2100 Bioanalyzer (Santa Clara, CA, USA). Sequencing was performed on the Illumina NovaSeq 6000 platform (San Diego, CA, USA), yielding 4,106,991 paired-end 150 bp reads (1.23 Gb; approximately 237× genome coverage). Raw reads were quality-trimmed using Trimmomatic v0.39 (5) and assembled into a genome using SPAdes v4.0.0 (6) under isolate mode, resulting in a genome of 122 contigs with a total length of 5,191,762  bp, an N*50* of 207,685  bp, and a GC content of 51.86%. Species identification was conducted using FastANI v1.34 (7), with strain 142109 sharing 99.94% average nucleotide identity with *Salmonella enterica* type strain ATCC 13883^T^ (accession no. CP065718.1). Genome annotation was performed using PGAP v6.10 (8), and a total of 4,926 protein-coding genes were identified. Antimicrobial resistance genes and serotype were predicted using AMRFinderPlus v4.0.22 (9) and SeqSero2 v1.3.1 (10), respectively. Virulence determinants were identified using BLASTn against the core *Salmonella* virulence gene data set provided by VFDB (11). Default settings were applied for all bioinformatics tools unless otherwise specified.

The strain was identified as *Salmonella enterica* serovar Typhimurium, with a predicted antigenic profile of 4:i:1,2. Comparison against public databases revealed multiple virulence-associated genes (Table 1) and antimicrobial resistance genes. These included aminoglycoside resistance genes (*aac (3)-IVa*, *aph(3')-Ia*, *aph (4)-Ia*, and *rmtB1*), the β-lactamase gene *bla*_TEM-1_, the carbapenem resistance gene *bla*_NDM-5_, the bleomycin resistance gene *ble*, the phenicol resistance gene *cmlA1*, the lincosamide resistance gene *lnu(F*), the quinolone resistance gene *qnrS1*, sulfonamide resistance genes (*sul1*, *sul2*, and *sul3*), and the trimethoprim resistance gene *dfrA12*. Antimicrobial susceptibility testing using the Vitek II automated microbiology system (bioMérieux, Marcy-l’Étoile, France) confirmed its resistance to levofloxacin (MIC ≥8  mg/L), cefotaxime (MIC ≥64  mg/L), imipenem (MIC ≥16  mg/L), and meropenem (MIC ≥16  mg/L).

**TABLE 1:** Virulence genes predicted in *Salmonella enterica* serovar Typhimurium strain 142109

Category/system	Detected genes
Adherence	
Agf (thin aggregative fimbriae)	*csgA, csgB, csgC, csgD, csgE, csgF, csgG*
Lpf (long polar fimbriae)	*lpfA, lpfB, lpfC, lpfD, lpfE*
Type 1 fimbriae	*fimC, fimD, fimF, fimH, fimI*
MisL	*misL*
RatB	*ratB*
SinH	*sinH*
Antimicrobial activity	
Mig-14	*mig-14*
Effector delivery system	
SPI-1 encoded T3SS	*orgA, orgC, steA, steB*
SPI-2 encoded T3SS	*gogB, pipB, pipB2, sifB, sopD2, sscA, sscB, sseI/srfH, sseK1, sseK2, sseL, steC*
TTSS (SPI-1 encoded)	*avrA, invA, invB, invC, invE, invF, invG, invH, invI, invJ, orgB, prgH, prgI, prgJ, prgK, sicA, sicP, sipA/sspA, sipB/sspB, sipC/sspC, sipD, slrP, sopA, sopB/sigD, sopD, sopE2, spaO, spaP, spaQ, spaR, spaS, sptP*
TTSS (SPI-2 encoded)	*sifA, spiC/ssaB, ssaC, ssaD, ssaE, ssaG, ssaH, ssaI, ssaJ, ssaK, ssaL, ssaM, ssaN, ssaO, ssaP, ssaQ, ssaR, ssaS, ssaT, ssaU, ssaV, sseA, sseB, sseC, sseD, sseE, sseF, sseG, sseJ, sspH2*
Nutritional/metabolic factor	
MgtBC	*mgtB, mgtC*
Stress survival	
SodCI	*sodCI*

## Data Availability

The genome assembly of *Salmonella enterica* serovar Typhimurium strain 142109 has been deposited in GenBank under the accession number JBNGEL000000000. The raw sequencing reads have been submitted to the Sequence Read Archive under accession number SRR33281733.
